# Extrapolation of Ventricular Activation Times From Sparse Electroanatomical Data Using Graph Convolutional Neural Networks

**DOI:** 10.3389/fphys.2021.694869

**Published:** 2021-10-18

**Authors:** Felix Meister, Tiziano Passerini, Chloé Audigier, Èric Lluch, Viorel Mihalef, Hiroshi Ashikaga, Andreas Maier, Henry Halperin, Tommaso Mansi

**Affiliations:** ^1^Pattern Recognition Lab, Friedrich-Alexander University, Erlangen, Germany; ^2^Digital Technology and Innovation, Siemens Healthineers, Erlangen, Germany; ^3^Digital Technology and Innovation, Siemens Healthineers, Princeton, NJ, United States; ^4^Cardiac Arrhythmia Service, Johns Hopkins University School of Medicine, Baltimore, MD, United States

**Keywords:** deep learning, graph convolutional networks, cardiac computational modeling, electroanatomic mapping, sparse measurements

## Abstract

Electroanatomic mapping is the gold standard for the assessment of ventricular tachycardia. Acquiring high resolution electroanatomic maps is technically challenging and may require interpolation methods to obtain dense measurements. These methods, however, cannot recover activation times in the entire biventricular domain. This work investigates the use of graph convolutional neural networks to estimate biventricular activation times from sparse measurements. Our method is trained on more than 15,000 synthetic examples of realistic ventricular depolarization patterns generated by a computational electrophysiology model. Using geometries sampled from a statistical shape model of biventricular anatomy, diverse wave dynamics are induced by randomly sampling scar and border zone distributions, locations of initial activation, and tissue conduction velocities. Once trained, the method accurately reconstructs biventricular activation times in left-out synthetic simulations with a mean absolute error of 3.9 ms ± 4.2 ms at a sampling density of one measurement sample per cm^2^. The total activation time is matched with a mean error of 1.4 ms ± 1.4 ms. A significant decrease in errors is observed in all heart zones with an increased number of samples. Without re-training, the network is further evaluated on two datasets: (1) an in-house dataset comprising four ischemic porcine hearts with dense endocardial activation maps; (2) the CRT-EPIGGY19 challenge data comprising endo- and epicardial measurements of 5 infarcted and 6 non-infarcted swines. In both setups the neural network recovers biventricular activation times with a mean absolute error of less than 10 ms even when providing only a subset of endocardial measurements as input. Furthermore, we present a simple approach to suggest new measurement locations in real-time based on the estimated uncertainty of the graph network predictions. The model-guided selection of measurement locations allows to reduce by 40% the number of measurements required in a random sampling strategy, while achieving the same prediction error. In all the tested scenarios, the proposed approach estimates biventricular activation times with comparable or better performance than a personalized computational model and significant runtime advantages.

## 1. Introduction

Ventricular tachycardia (VT) is a serious cardiac condition that may lead to hemodynamic collapse and sudden cardiac death (John et al., 2012). It is commonly observed in patients after myocardial infarction, which exhibit heterogeneous scar distributions. In particular, channels of surviving slow-conductive tissue, so-called “border zone” (BZ), promote electrical wave re-entry (Al-Khatib et al., 2018).

Catheter-based radiofrequency ablation is an established treatment for VT, which aims at eliminating re-entrant circuits responsible for sustaining VTs. This procedure is, however, only successful in about 50% of infarct-related VT cases (John et al., 2012). The efficacy of this procedure is directly linked to the precise delineation of the arrhythmogenic substrate and the identification of re-entry origin (John et al., 2012; Al-Khatib et al., 2018).

Imaging modalities such as late gadolinium enhanced magnetic resonance imaging (LGE-MRI) or computed tomography can provide information about the extent of the arrhythmogenic substrate. However, assessing the electrophysiological behavior and origin of wave re-entry may not be possible solely from images due to the inability to relate image intensity to precise voltage and activation time distributions. Electroanatomical mapping (EAM) is therefore conventionally used to inspect the arrhythmogenic substrate and to identify the origin of wave re-entry (John et al., 2012). Despite being the gold standard, it poses practical challenges (Josephson and Anter, 2015). For instance, persistent episodes of scar-related VT could lead to hemodynamic collapse, which prohibits the acquisition of high-resolution maps that are necessary to determine the origin of wave re-entry and abnormal wave fronts related to scar and slow-conductive channels (John et al., 2012; Al-Khatib et al., 2018). In addition, EAM yields measurements on the tissue surface only, thus preventing the determination of electrical activity within the myocardium (Ashikaga et al., 2007).

Methods to obtain high-resolution maps from sparse measurements commonly rely on interpolation with linear or radial basis functions. The accuracy and quality of the interpolation method is hereby dependent on the given EAM, as has been investigated by Sanromán-Junquera et al. for approaches like thin plate splines and support vector machines (Sanromán-Junquera et al., 2015). Since these methods neglect the underlying surface geometry and uncertainties in electrode recordings, Coveney et al. proposed a probabilistic interpolation of atrial EAMs based on Gaussian Markov random fields (Coveney et al., 2020). Aiming at incorporating physical priors into the interpolation method, Costabal et al. investigated the use of physics-informed neural networks, which significantly outperformed linear and Gaussian process interpolation (Sahli Costabal et al., 2020). This work was further extended to account for the tissue anisotropy and to obtain information of prevalent fiber directions (Grandits et al., 2021).

While these methods may provide accurate high-resolution maps from sparse measurements, their accuracy on capturing intramural activation times, specifically for slow conductive border zone, has not been investigated. Hence, they focus mainly on atrial EAM reconstruction since the measurements provide only surface information and modeling the atrium as a triangular mesh is a viable modeling option due to the low thickness to diameter ratio (Sahli Costabal et al., 2020).

To obtain coarse estimates of intramural abnormalities in VT cases, a recent study suggests simultaneous mapping of both endocardium and epicardium (Tung et al., 2020), which comes at the price of longer and riskier mapping procedures. An alternative solution may be realized by computational models of cardiac electrophysiology. By combining imaging information and (non-)invasive measurements, such mathematical models of the electrical wave propagation already proved to be promising approaches to reduce uncertainties in tissue conductivity estimates from EAMs (Wallman et al., 2014), to study VT mechanisms (Martinez-Navarro et al., 2019), and to predict VT ablation outcome (Cedilnik et al., 2018; Prakosa et al., 2018; Corral-Acero et al., 2020). Critical for accurate predictions is the selection of modeling assumptions such as the choice of the cell model or the fiber model. In addition, the model requires a robust and accurate personalization scheme to estimate local tissue properties from measurements. For instance, Pheiffer et al. proposed a personalization scheme of local tissue conductivities from a left-ventricular endocardial EAM using backpropagation of errors along the wave propagation paths and gradient descent to tune the tissue properties (Pheiffer et al., 2017). Even though standard personalization methods are able to match the measured data, their accuracy is dependent on a careful selection of the modeling parameters and the boundary conditions, e.g., the choice of electrical propagation origin.

This work investigates a deep learning based reconstruction of left endocardial activation maps from a set of sparse measurements as well as the extrapolation of approximate activation times in the biventricular myocardium. To this end, we propose the use of a graph convolutional neural network on a tetrahedral discretization of cardiac anatomy derived from MR images. Graph convolutional layers leverage feature information of vertices within local neighborhoods defined on a graph. By stacking these convolutional layers, the network is able to propagate information over a large receptive field. Since no ground truth information of intramural activation times is available, a computational model of cardiac electrophysiology with varying physical parameters and boundary conditions is used to provide synthetic ground truth information.

This research directly builds on our previous work (Meister et al., 2021) with the following new contributions:

- To train the neural network, the previous work uses a synthetic dataset with simplified and homogeneously distributed scar and border zone. In particular, per simulation one of the 17 left ventricular AHA regions is set to be either non-conductive or weakly conductive, which limits the generalization to the complex distributions observed *in vivo*. In this work, more complex scar and border zone geometries as well as variations in the initial activation locations are used to better reflect the real world. Increasing the local variations within the ground truth activation maps helps guide the network to make localized predictions, thus improving the quality of reconstructed activation times in the pre-clinical datasets.
- Evaluation of the proposed method's ability to reconstruct high-resolution endocardial activation maps from sparse measurements on four porcine cases with high-resolution electroanatomical maps.
- Evaluation of the proposed method's ability to reconstruct epicardial activation times on the CRT-EPIGGY19 challenge data comprising 11 porcine cases (6 non-infarcted, 5 infarcted) with both endocardial and epicardial measurements.
- Evaluation of the proposed method's ability to support the mapping procedure by suggesting new sampling locations.

The manuscript is structured as follows: Section 2 introduces the details of our proposed pipeline, as well as the Eikonal model of cardiac electrophysiology which was used to generate the training and testing data. Since the computational model further acts as a baseline method when personalized to the sparse data, a personalization strategy is introduced in the same section. In section 3, results on both synthetic and real-world datasets are presented. The accuracy under various subsampling ratios is quantified and compared against the personalized computational model. Section 4 discusses the results and section 5 concludes the manuscript.

## 2. Materials and Methods

### 2.1. Graph Convolutional Estimation of Biventricular Local Activation Times

#### 2.1.1. Overview of Graph Convolutional Processing Pipeline

**Figure 1:**
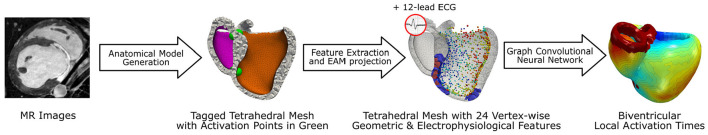
Illustration of the proposed deep learning based pipeline to estimate biventricular local activation times. In a first step the cardiac anatomy including scar (red area) and border zone distribution (blue area) is segmented from MR images and discretized by a tetrahedral mesh. The sparse measurement cloud of endocardial activation times (colored spheres) is manually registered and mapped to the mesh. A graph convolutional neural network is using the mesh and vertex-wise features to estimate the local activation times in the entire biventricular domain.

To estimate the biventricular local activation times, this work proposes a deep learning based pipeline (see Figure 1), which uses as input MR images, a routinely acquired 12-lead electrocardiogram (ECG), and a sparse measurement cloud of left endocardial activation times. First, the biventricular cardiac anatomy is segmented from the MR images. In this work, we use a manual approach to capture the specific features of the swine cardiac anatomies used for validation. Furthermore, scar and border zone are manually annotated from the same images. In a second step, a tetrahedral anatomical model is constructed by adding a rule-based fiber model and by tagging different tissue classes (Mansi et al., 2019). In addition, a set of four standard initial activation points is added: basal and apical on the left and right septal wall, respectively (see Figure 1).

Next, the sparse measurement point cloud is manually registered by an electrophysiologist since the recorded catheter locations and the anatomical model do not share a common coordinate system. Voltage measurements are used to guide the alignment process, because low voltage areas correlate with scar masks derived from MR images (Nakahara et al., 2011). Outliers in the activation time measurements are removed using a threshold of 1.5 standard deviations away from the mean of local activation times. Geometric and electrophysiological features are extracted from the cardiac geometry and the ECG traces (see section 2.1.3), respectively. A graph convolutional neural network trained on synthetic data is processing the input features to estimate the local activation times in the biventricular domain (see section 2.1.4).

#### 2.1.2. Definition of Graph Convolution

Graph convolutional neural networks are chosen in this work since they are able to learn from graph structured data, i.e., the tetrahedral computational domain. Their usage naturally adapts to the problem of learning the electrophysiology since the wave propagation is heavily influenced by the structure of the cardiac conduction system. In this work, the biventricular heart geometry is represented by a mesh with linear tetrahedral elements (Kayvanpour et al., 2015). More generically, the mesh is expressed as an undirected graph G=(V,E,X). The graph is composed of a set of *N* vertices $V=\left\{v_{0},\ldots ,v_{N}\right\}$, vertex-wise *D*-dimensional feature vectors summarized in a feature matrix **X** ∈ **R**^*N*×*D*^, and a set of edges E⊆V×V corresponding to the edges of the tetrahedral mesh. In this work, only undirected edges are considered. We define a vertex *v*_*i*_'s neighborhood $N\left(v_{i}\right)=\left\{v_{j}\;|\;\text{iff}e_{ij}\in E\right\}$ as all 1-hop connected vertices.

The proposed network uses the so-called GraphSAGE layers, a generalized formulation of message passing graph convolutions (Hamilton et al., 2017). Each layer *l* acts on the local neighborhoods of all vertices independently, while sharing learnable feature transformations between them. Given the representation $h_{i}^{l}$ of a vertex *v*_*i*_ at layer *l*, with $h_{i}^{0}\in \text{X}$ being the initial vertex feature, GraphSAGE first computes a neighborhood aggregate.


(1) $$\begin{array}{l} h_{N\left(v_{i}\right)}^{\left(l+1\right)}=\text{aggr}\left(h_{j}^{l},j\in N\left(v_{i}\right)\right) \end{array}$$

with “aggr” denoting any permutation-invariant aggregation function such as mean, max, and sum. In this work mean aggregation is used. Each vertex representation is then updated according to


(2) $$\begin{array}{l} h_{i}^{\left(l+1\right)}=\sigma \left(\text{W}\cdot \left(h_{i}^{l}\;||\;h_{N\left(v_{i}\right)}^{\left(l+1\right)}\right)\right) \end{array}$$

where σ is a non-linear activation function, **W** a learnable weight matrix, which is shared across all vertices, and || denotes the concatenation of *v*_*i*_'s features $h_{i}^{l}$ and the neighborhood aggregate $h_{N\left(v_{i}\right)}^{\left(l+1\right)}$.

#### 2.1.3. Feature Description

**Figure 2:**
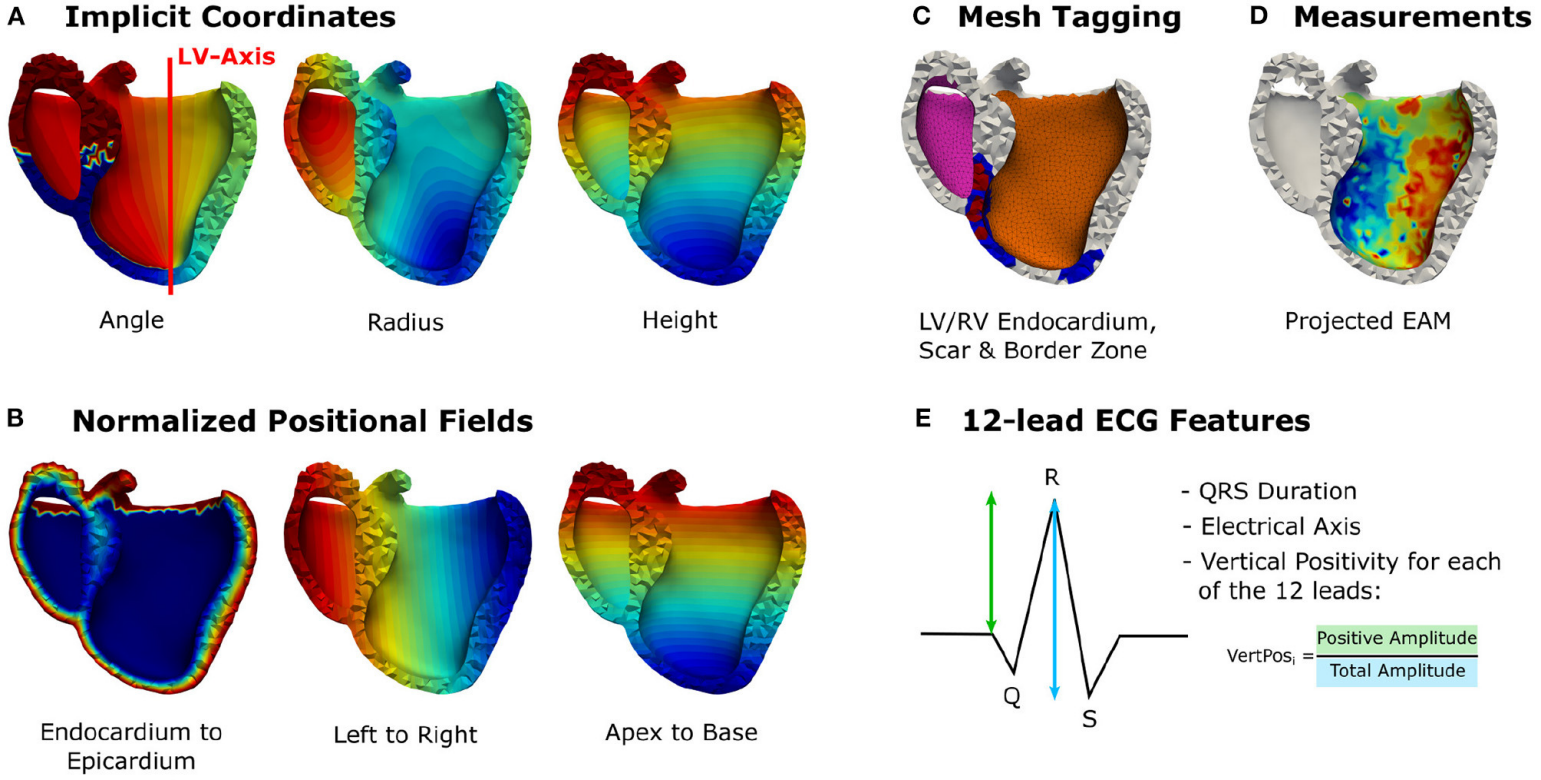
Illustration of the incorporated vertex-wise features: **(A)** Positional encoding of vertex positions in a cylindrical coordinate system. **(B)** Additional relative positional encodings. **(C)** Categorical features denoting vertices belonging to the left or right endocardium (pink, orange), scar (red), or border zone (blue). **(D)** The projected electroanatomical measurements. **(E)** Fourteen features extracted from the 12-lead ECG traces.

The feature matrix **X** comprises per vertex a total of 24 geometric and electrophysiological features (see Table 1). A primary geometric feature is a descriptor of the vertex position within the mesh. If 3D cartesian coordinates are used, the training dataset will require significant augmentation to cover the space of all possible affine transformations and make the network generalize to arbitrarily oriented hearts. Therefore, the vertex position is described in a local coordinate system that is consistent between different heart geometries, i.e., a cylindrical coordinate system defined with respect to the left ventricular axis (see Figure 2A). In addition to angle, radius, and height features, [0, 1]-normalized fields describing the relative position between apex and base, left and right ventricle, and endocardium and epicardium are added (see Figure 2B). Furthermore, three categorical features are added, which prescribe a value of 1 to vertices belonging to a specific tissue class and 0 otherwise. Two categorical features capture vertices belonging to the left or right endocardium, respectively (see Figure 2C). The third feature takes the value 1 if the vertex is part of healthy tissue and 0 if it is part of border zone. Vertices belonging to scar tissue are removed as we consider them as not depolarizing.

**Table 1:** An overview of all 24 geometric and electrophysiological features.

**Feature ID**	**Feature name**
**Geometric features**	
1	Angle
2	Radius
3	Height
4	Relative position: apex to base
5	Relative position: left to right
6	Relative position: endocardium to epicardium
7	Tissue health: 1 = healthy tissue, 0 = border zone
8	Categorical feature: 1 = Left endocardium, 0 = otherwise
9	Categorical feature: 1 = Right endocardium, 0 = otherwise
**Electrophysiological features**	
10	EAM measurement
11	QRS duration
12	Electrical axis
13-24	Vertical positivities: Relative amplitude for each lead of the 12-lead ECG

The main electrophysiological features are the sparse endocardial measurements. A default value of –1 is prescribed for all vertices that do not have any measurements available (see Figure 2D). Additionally, features are extracted from the 12-lead ECG traces. These features are stored per vertex since the network is making individual predictions for all vertices, while sharing the network parameters for all predictions. This work uses the QRS duration, the electrical axis, and 12 features describing the positiveness of the QRS complex amplitude per ECG lead in percent (see Figure 2E). All features except the endocardial measurements are normalized to the [0, 1]-interval using the bounds of the training data. To consistently normalize the ground truth and endocardial measurements, we normalize the sparse measurements using the training data bounds of the target activation times. At test time, the training bounds are used to normalize the input features.

#### 2.1.4. Graph Convolutional Network Architecture

**Figure 3:**
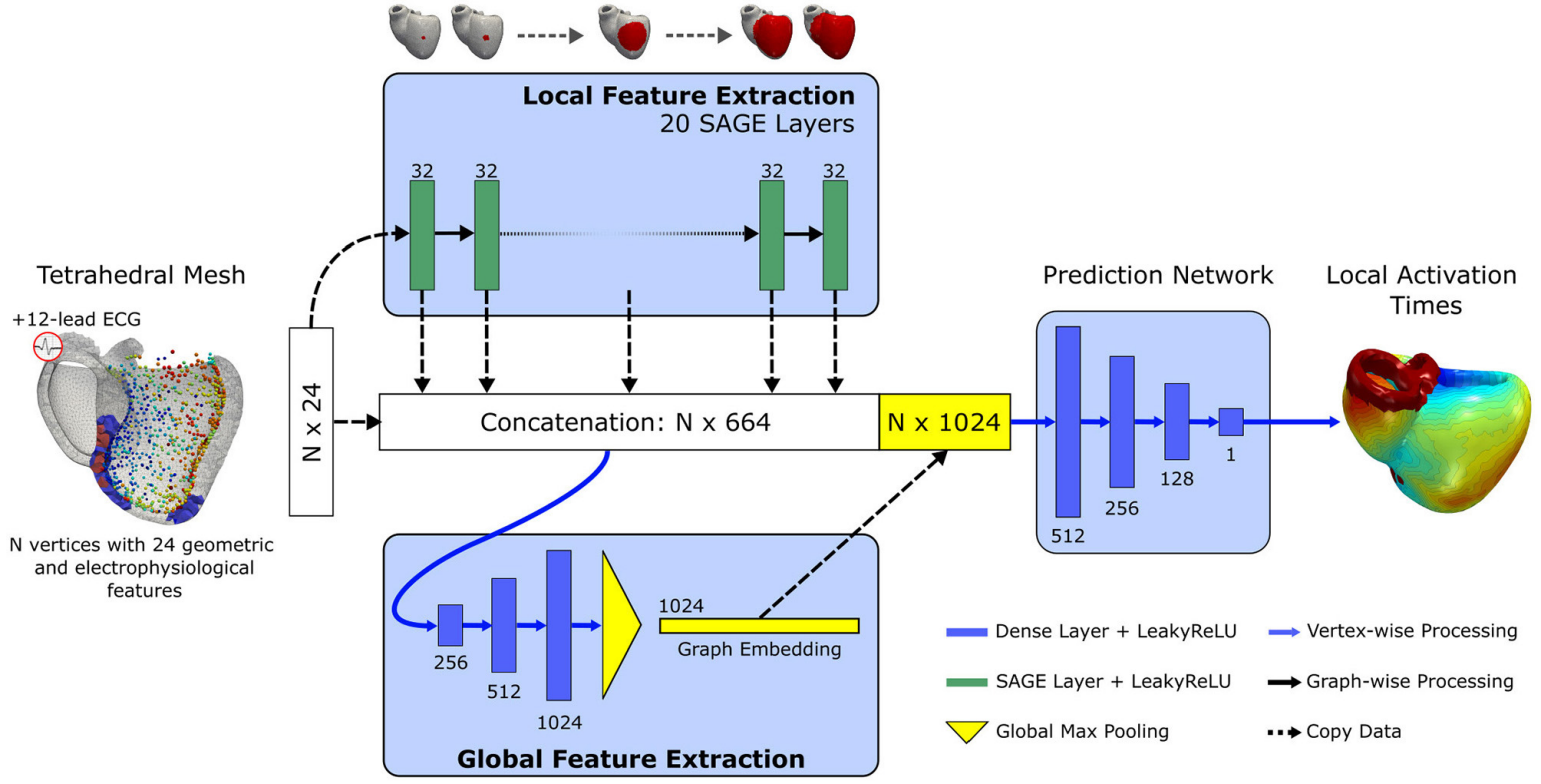
Illustration of the proposed graph convolutional network architecture. Input is a tetrahedral mesh representing the biventricular anatomy. Per vertex, 24 geometric and electrophysiological features are extracted. First, a series of 20 GraphSAGE convolutional layers with 32 units and leaky rectified linear activation are applied to extract local features over an increasing receptive field. The output of each layer as well the input features are concatenated. The concatenated feature vector is further processed by a global feature extractor, which applies three fully connected layers of increasing size and a final global max pooling. The pooled feature vector is appended to the concatenated feature vector. Local activation times in the entire biventricular geometry are estimated by processing the combined feature vectors with three non-linear fully connected layers and a final linear transformation.

The proposed neural network builds upon a well-established architecture for deep learning based point cloud processing, called PointNet (Qi et al., 2017). The neural network is processing all points of the input mesh independently while sharing the learnable parameters. The PointNet architecture comprises a local feature extractor, a global feature extractor, and a point-wise prediction network. The structural transformer layers in the local feature extractor are replaced by a series of GraphSAGE layers (see section 2.1.2) to allow the exchange of information over neighborhoods of increasing size via message passing. Each vertex of the mesh may require information from different receptive fields, e.g., a vertex on the endocardium may have a measurement point in the direct neighborhood while a vertex on the epicardium requires information from multiple hops away. To enable the network to learn from different receptive fields, the output of each layer of the local feature extractor as well as all input features are concatenated per vertex to form a large local feature matrix. Each row equals the local feature vector of a specific vertex in the mesh. A series of fully connected layers with leaky rectified linear activation function and global max pooling is applied to extract a global feature vector per mesh. The global features are appended to the local feature matrix and further processed by fully connected layers to estimate the local activation time for each vertex. An illustration of our adapted architecture can be seen in Figure 3.

#### 2.1.5. Implementation

The proposed architecture is implemented using PyTorch (version 1.8) and PyTorch Geometric (version 1.6.3) (Fey and Lenssen, 2019; Paszke et al., 2019). The number of layers as well as their sizes are chosen empirically using a small subset of training examples and using the PointNet architecture details as guidance (Qi et al., 2017). For the local feature extractor we choose 20 GraphSAGE layers, each with 32 units and leaky rectified linear activation. No improvement in performance has been observed when using more than 20 graph convolutional layers. For the global feature extractor we select three fully connected layers with 256, 512, and 1,024 units and leaky rectified linear activation. The final prediction network comprises three fully connected layers (512, 256, and 128 units, respectively) with leaky rectified linear activation and a final fully connected layer with one linear unit. The network is trained in a multi-gpu setup comprising 8 NVIDIA Tesla V100 graphics cards for 2,000 epochs. The Adam optimizer (Kingma and Ba, 2014) with default parameters and an initial learning rate of 5 × 10^−4^ is chosen to optimize the network parameters. To stabilize the training, the learning rate is reduced by 20% every 25 epochs. For the loss function we choose


(3) $$\begin{array}{l} L=L_{\text{LAT}}+L_{\text{TAT}} \end{array}$$

where *L*_LAT_ is the weighted mean squared error loss


(4) $$\begin{array}{l} L_{\text{LAT}}=\frac{1}{N}\underset{i}{\sum }\alpha_{i}{\left(y_{i}-{ŷ}_{i}\right)}^{2} \end{array}$$

with *N* denoting the number of vertices with ground truth local activation time *y*_*i*_, and ŷ_*i*_ the predicted activation time. α_*i*_ is a weighting factor, which we set to two for vertices belonging to the left endocardium to put more emphasis on making accurate predictions in this region. A weighting of α_*i*_=1 is used for all other vertices. To guide the network to match the total activation time (TAT), we apply an additional regularization *L*_TAT_ defined by


(5) $$\begin{array}{l} L_{\text{TAT}}={\left(\left(\max\hat{Y}-\min\hat{Y}\right)-\left(\max Y-\min Y\right)\right)}^{2} \end{array}$$

with Y the set of ground truth activation times and $\hat{Y}$ the set of predicted activation times. Since the network parameters are shared between the predictions for all vertices, one cannot guarantee that the trained graph convolutional network is able to match the local activation times in the vertices with measurements. To emphasize a correct fit in the measurements, a rescaling is applied to the trained network as a postprocessing step. We aim to match the range of activation time measurements on the left endocardium. Given the set of measurements A and the predictions $\hat{A}$ in these vertices we scale the prediction according to,


(6) $$\begin{array}{l} {\hat{Y}}^{*}=\left(\hat{Y}-\left(\min\hat{A}-\min A\right)\right)\cdot \frac{\left(\max A-\min A\right)}{\left(\max\hat{A}-\min\hat{A}\right)} \end{array}$$

### 2.2. Personalized Graph-Based Computational Model of Cardiac Electrophysiology

Since ground truth intramural activation times are commonly not available from *in-vivo* interventions, this work relies on a synthetic dataset generated by a fast graph-based electrophysiological model (Pheiffer et al., 2017). The physiological priors of the cardiac anatomy are expected to be known. For instance, an anatomical model with a rule-based fiber model and a pre-defined set of initial activation locations is assumed to be available (see section 2.1.1 for comparison). The local activation time *y*_*i*_ for every vertex *v*_*i*_ of the tetrahedral mesh representing the cardiac anatomy is estimated by computing the shortest path to a set of activation points via the Dijkstra algorithm. To incorporate tissue anisotropy, generalized edge weights considering the fiber direction are computed. For an edge between vertices *v*_*i*_ and *v*_*j*_, the generalized edge weight *w*_*ij*_ is calculated as


(7) $$\begin{array}{l} w_{ij}=l_{ij}/c_{ij} \end{array}$$

with the edge conduction velocity *c*_*ij*_ in mm/s that is computed by a linear interpolation of the conduction velocities at vertex *v*_*i*_ and *v*_*j*_. The effective edge length *l*_*ij*_ is computed as


(8) $$\begin{array}{l} l_{ij}=\sqrt{\left({\vec{e}}_{ij}^{T}\text{D}{\vec{e}}_{ij}\right)} \end{array}$$

where ${\vec{e}}_{ij}$ is the edge vector between the two vertices. The anisotropy tensor **D** is computed from the fiber direction ${\vec{f}}_{ij}$ along the edge and anisotropy ratio *r* according to


(9) $$\begin{array}{l} \text{D}=\left(1-r\right){\vec{f}}_{ij}{\vec{f}}_{ij}^{T}+r\text{I} \end{array}$$

where **I** is the identity matrix. In this work, an anisotropy ratio *r* of 0.3 is used and fibers are modeled by a rule-based fiber model (Kayvanpour et al., 2015; Mansi et al., 2019).

For a specific path connecting an initial activation point *v*_init_ to a vertex *v*_*i*_ with measurements, the activation time at *v*_*i*_ can be expressed by


(10) $$\begin{array}{l} {ŷ}_{i}=t_{\text{init}}+\underset{k}{\sum }w_{k} \end{array}$$

where *t*_init_ denotes the time when the depolarization starts at *v*_init_ and the set {*w*_1,…,*K*_} represents the generalized edge weights along this path. In a second step, intracellular transmembrane potentials are approximated using a rule-based approach (Zettinig et al., 2014). For a given time *t*, the intracellular potential ϕ_*i*_ of a vertex *v*_*i*_ is computed from its local activation time ŷ_*i*_ according to


(11) $$\phi_{i}({\hat{y}}_{i},t)=\{\begin{array}{ll} -70\text{ mV}, & \text{if }{\hat{y}}_{i}>t.\\ +30\text{ mV}, & \text{if }{\hat{y}}_{i}\leq t. \end{array}$$

**Figure 4:**
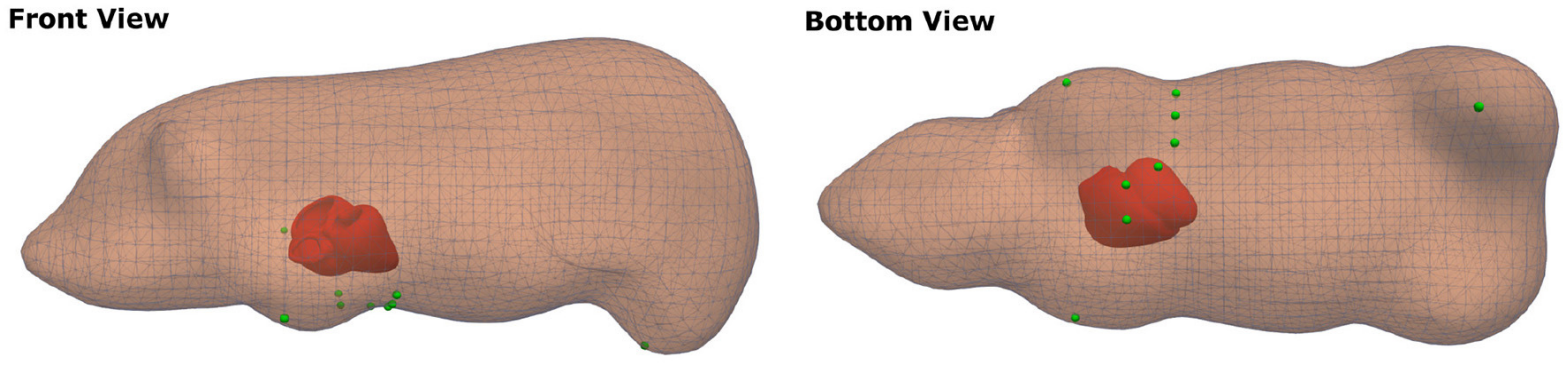
Visualization of the swine torso template with ECG lead placement (green markers), which were used for the computation of synthetic ECGs.

Following the description in Zettinig et al. (2014) and Mansi et al. (2019), vertex-wise extracellular transmembrane potentials are computed, which are then mapped to a triangulation of the torso with ECG electrode positions annotated (see Figure 4 for reference). From the mapped body surface potentials a 12-lead ECG is calculated.

#### 2.2.1. Model Personalization Description

The electrophysiological model is also used as a baseline in this study. To this end, we require a personalization scheme that finds the best set of edge weights that explains the data, i.e., the sparse electroanatomical map as well as the routinely acquired 12-lead ECG. This work leverages the approach proposed by Pheiffer et al. (2017). The first step comprises a global optimization of homogeneous tissue conduction velocities. The cardiac anatomy is hereby divided into five tissue classes: the myocardium, the left endocardial Purkinje system, the right endocardial Purkinje system, border zone, and scar. The left and right endocardial Purkinje system comprises all vertices within a 3 mm thick layer below the respective surface. Vertices representing scar and border zone are annotated from the MR image segmentations. All other vertices are considered to belong to the myocardium. Scar is chosen to be non-conductive (0 mm/s). A conduction velocity for each of the other four tissue classes is optimized to match the ECG traces, specifically the QRS duration and electrical axis, by using the BOBYQA algorithm (Powell, 2009). To reduce the number of parameters to be estimated, we constrain the border zone conductivity to be 50% of the myocardial conductivity.

A second step refines the edge weights to match the sparse measurements (Pheiffer et al., 2017). The objective function in this case is the mean squared error loss over the *M* vertices with measurements


(12) $$\begin{array}{l} L_{\text{MSE}}=\frac{1}{M}\underset{i}{\sum }{\left(y_{i}-{ŷ}_{i}\right)}^{2} \end{array}$$

where *y*_*i*_ denotes the activation time measurement for vertex *v*_*i*_. Similarly, ŷ_*i*_ corresponds to the simulated activation time at vertex *v*_*i*_. The estimated activation time for any vertex in the tetrahedral mesh is dependent on the edge weights along the shortest path to the associated activation point and the initial activation time (see Equation 10). This setting is similar to neural networks where the output is dependent on the parameters of the hidden layers and the input to it. Backpropagation and gradient descent is therefore used to fine-tune the edge weights of paths connecting the vertices with measurements with their shortest-path-connected activation points.

The gradient descent update rule of an edge weight writes


(13) $$\begin{array}{l} w_{k}^{t+1}=w_{k}^{t}-\gamma g \end{array}$$

where *t* denotes the current iteration number, γ the step size, and *g* the gradient. We seek gradients $g=\frac{\partial L}{\partial w_{k}}$ that minimize Equation 12. The backpropagation algorithm yields gradients along the entire path by recursively applying the chain rule from the end to the start of the path. This can be formulated as


(14) $$\begin{array}{l} \frac{\partial L}{\partial w_{k}}=\frac{\partial L}{\partial {ŷ}_{i}}\frac{\partial {ŷ}_{i}}{\partial w_{k}} \end{array}$$

The gradient at the path end can be derived from the Equation (12) by


(15) $$\begin{array}{l} \frac{\partial L}{\partial {ŷ}_{i}}=-\frac{2}{M}\left(y_{i}-{ŷ}_{i}\right) \end{array}$$

and the gradient of the activation time with respect to any edge weight from Equation (10) by


(16) $$\begin{array}{l} \frac{\partial {ŷ}_{i}}{\partial w_{k}}=1 \end{array}$$

Since there is an optimization path associated with each measurement point, an edge might be traversed several times. We accumulate gradients before updating the weights with Equation (13).

## 3. Results

### 3.1. Data Generation

The first cohort comprises 15 swine datasets. Each dataset consists of MR images, 12-lead ECG traces (CardioLab, GE Healthcare) and a left endocardial contact map (Ensite Velocity System, St. Jude Medical). The EAM was recorded with a standard irrigated radiofrequency ablation catheter (FlexAbility, Abbott) and captured the intrinsic cardiac activation after left bundle branch block (LBBB) induction. Due to the limited amount of data and the absence of ground truth information of intramural local activation times, the computational model from section 2.2 was incorporated to build a synthetic training set.

First, 11 of the 15 swine datasets with the fewest EAM measurements were selected. Segmentations of the cardiac biventricular anatomy were extracted from the MR images. These segmentations were then used to construct a statistical shape model. To this end, the triangular segmentations were rigidly aligned using point correspondences. The principal component analysis was applied to extract a mean model and the modes of variation. In total, 200 geometries were sampled from the statistical shape model using only the five most informative eigenvectors explaining more than 80% of the variance. Twenty models were discarded due to implausible geometries. The “Computational Geometric Algorithms Library” (CGAL) was used to create tetrahedral meshes with a mean edge length of approximately 2.5 mm (The CGAL Project, 2021). To simulate synthetic ECGs, a generic swine torso with standard ECG placement (see Figure 4 for reference) was first manually aligned to match the MR images in one case and then automatically registered to the other anatomical models using rigid registration.

For each of the 180 synthetic geometries, a total of 100 simulations were computed by varying the initial activation, the tissue conduction velocities, and the scar and border zone distribution. The first 50 simulations per geometry were computed using a set of four activation points located on the septum: left and right, basal and apical (see Figure 1 for reference). The other 50 simulations were generated using three randomly placed activation points on the left ventricular endocardium. To model the arrhythmogenic substrate, we randomly select for each simulation the number of scars (up to three). A random point on the left ventricle is then iteratively selected. Per point we randomly select two distance thresholds. The first threshold, ranging between 5 and 12.5 mm, will be used to denote all vertices within the given radius to be scar. The second threshold, a 3–10 mm margin around the scar, will define the border zone. To model more complex scar distributions with channels between them, the second or third scar will be placed within a 25 mm margin around the current scar distribution. Furthermore, vertices that were already assigned to border zone or scar will not be overwritten.

The conduction velocities were varied under the assumption of homogeneous conduction within five tissue classes. Scar was modeled with 0 mm/s. The general myocardium was modeled with a conduction velocity of *c*_Myo_ ∈ [250 mm/s, 750 mm/s]. The conduction velocity in the border zone was randomly varied to be *c*_BZ_ ∈ [100 mm/s, *c*_Myo_]. Furthermore, the left and right Purkinje systems (*c*_LV_ & *c*_RV_), each modeled by a 3 mm thick endocardial layer, have had conduction velocities that varied between 1,000 and 2,500 mm/s.

From the 18,000 simulations, we discarded all simulations with a simulated QRS duration greater than 200 ms, amounting to approximately 10% of all simulations, to stay within physiologically plausible ranges. The remaining simulations were randomly split by geometry into a training set (90%), a validation set (5%), and a test set (5%). In addition, the training dataset was augmented in each epoch by randomly subsampling the left endocardial ground truth using a random subsampling ratio between 10 and 100%.

### 3.2. Reconstruction of Biventricular Activation Times

After fitting the network to the training set, we first evaluated whether our proposed method can reconstruct the local biventricular activation times under unseen conditions. To this end, the network was applied to the 5% left out simulations from the synthetic database, which comprises 9 unseen geometries yielding a total of 870 unseen depolarization patterns. In particular, we subsampled the endocardial ground truth at various ratios (1, 2, 5, 10, 15, 20, and 25%) and had the network reconstruct the local activation times in the entire biventricular domain. We chose the mean absolute error (L1-error) to quantify the difference in local activation times for different tissue classes: the biventricular heart, the left endocardium, the left epicardium, and the border zone. In addition, we quantified the total activation time error, approximated by the range of the predicted or ground truth activation times.

The results as seen in Table 2 suggest that the network is able to leverage the endocardial information since the errors are decreasing for all tissue types when increasing the number of provided samples. Furthermore, the network is able to accurately reconstruct endocardial activation maps. Even in a setting of only 0.4 samples per cm^2^ (equal to a subsampling ratio of 2% and less than observed during training) the network reconstructs the complete endocardial ground truth with a mean absolute error of less than 5 ms. The mean absolute error rapidly drops to approximately 1 ms when increasing the number of samples beyond 2.1 samples/cm^2^. Similarly, we observe that the network is able to match the QRS duration effectively. Moreover, a significant decrease of the mean absolute errors for the border zone tissue is observed with an increased number of provided endocardial samples. This suggests that the network is able to infer to some extent the conductive property of the slow-conductive tissue, which may help in reducing ambiguities in the depolarization pattern.

**Table 2:** Mean absolute errors (± the standard deviation) in ms between the prediction and the synthetic ground truth activation time for different tissue types as well as the total activation time (TAT) at different subsampling ratios.

**Subsampling ratio**	**1%**	**2%**	**5%**	**10%**	**15%**	**20%**	**25%**
**∅ Samples / cm^**2**^**	**0.2**	**0.4**	**1.0**	**2.1**	**3.3**	**4.3**	**5.5**
TAT in ms	3.3 ± 3.7	2.1 ± 2.2	1.4 ± 1.4	1.2 ± 1.1	1.1 ± 1.2	1.1 ± 1.2	1.0 ± 1.1
Whole heart in ms	7.6 ± 7.4	5.7 ± 5.9	3.9 ± 4.2	3.3 ± 3.7	3.2 ± 3.7	3.1 ± 3.6	3.1 ± 3.6
LV Endo. in ms	7.3 ± 7.4	4.7 ± 5.4	2.4 ± 2.9	1.5 ± 1.9	1.3 ± 1.7	1.1 ± 1.4	1.0 ± 1.2
LV Epi. in ms	8.8 ± 8.2	6.7 ± 6.5	4.5 ± 4.5	3.9 ± 3.9	3.7 ± 3.8	3.7 ± 3.7	3.6 ± 3.6
Border zone in ms	9.4 ± 8.8	7.4 ± 7.3	5.3 ± 5.6	4.3 ± 4.6	4.0 ± 4.3	3.9 ± 4.2	3.7 ± 4.1

### 3.3. Reconstruction of High-Resolution Endocardial Maps From Sparse Measurements

In a second experiment the same network trained on the synthetic dataset is applied to the four swine datasets that were not used to construct the statistical shape model. For each of the four cases, scar and border zone were segmented from the MR images. The associated high-resolution electroanatomical map was manually registered by an electrophysiologist since the recorded catheter positions and the anatomical model do not share a common coordinate system. The resulting measurements were then mapped to all vertices on the endocardial surface of the tetrahedral meshes using nearest neighbor projection. The projected measurements were then randomly subsampled with the following ratios: 0.2, 0.4, 1.0, 2.1, 3.3, 4.3, 5.5, and 6.5 samples/cm^2^. The same samples were provided as input to three different methods for reconstructing the full endocardial measurement map: the graph convolutional neural network, the computational model, and a naive nearest neighbor projection. In the latter case, the raw EAM was first filtered to contain only the data points that would be projected to the subsampled points. Then, the reduced point cloud was again mapped to all vertices of the endocardial surface. The three methods were compared in terms of their mean absolute errors to reconstruct the ground truth high-resolution activation map.

**Figure 5:**
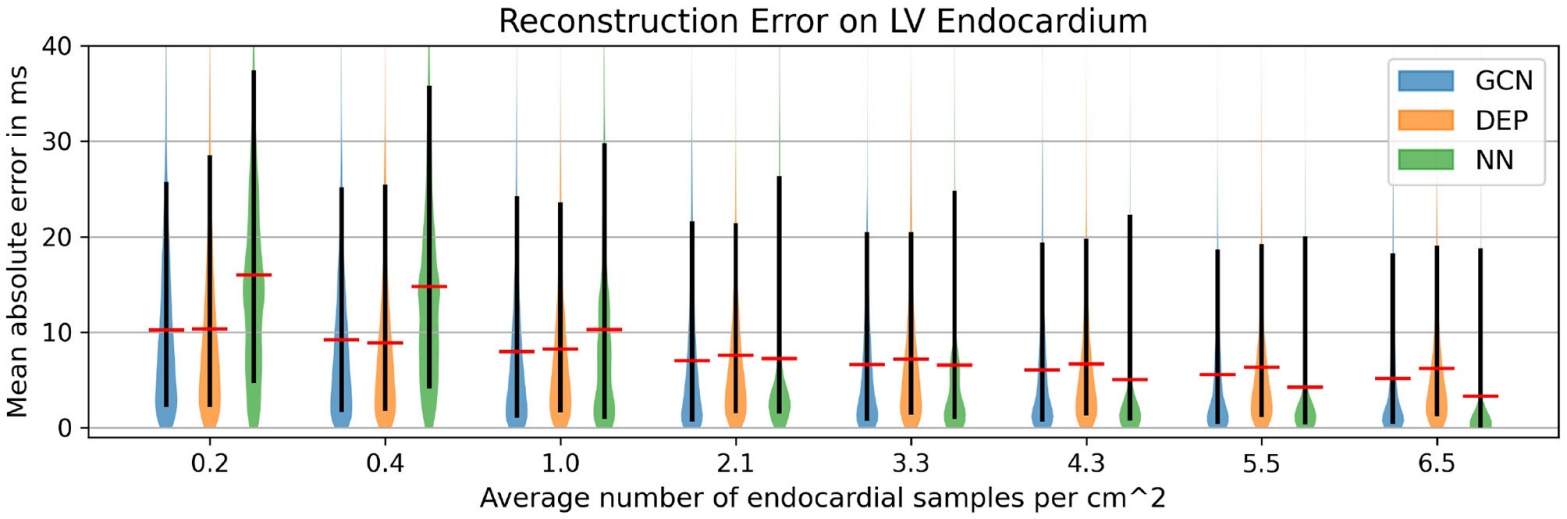
Mean absolute error for different subsampling ratios on our in-house cohort comprising four swine datasets with high-resolution endocardial EAMs. Comparison of the graph convolutional network (GCN), the personalized computational model (DEP), and a naive nearest neighbor projection (NN). The red bar denotes the mean, the black bar denotes the 15–95 percentiles.

**Figure 6:**
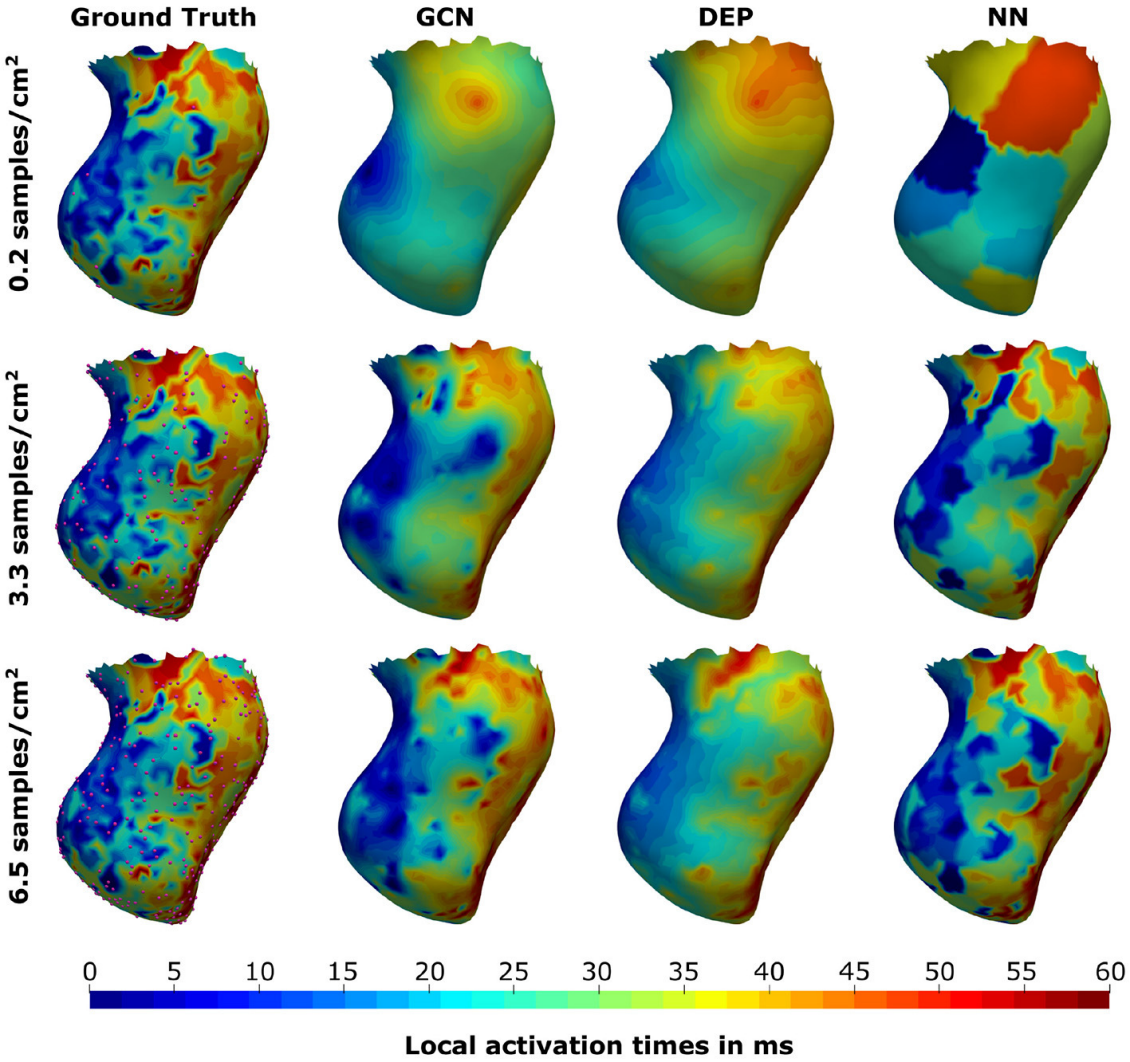
Illustration of the prediction results for the graph convolutional neural network (GCN), the personalized computational model (DEP), and the nearest neighbor projection method (NN) for different sampling ratios. Provided samples are highlighted by pink spheres.

As can be seen in Figure 5, both the graph convolutional network and the personalized computational model are producing significantly lower mean absolute errors for very low sampling ratios (<2.1 samples/cm^2^) compared to the projection method. All three methods improve substantially with increased sampling ratios. For larger sampling ratios it is expected that the projection method is outperforming the other two methods since the nearest neighbor projection may assign the same raw measurement point to multiple vertices on the endocardial surface, thus increasing the chances that a majority of the raw measurement points are used. When qualitatively comparing the results (see Figure 6), it can be observed that the computational model is producing a smooth activation map adherent to the modeling priors and moderately affected by the sampling points. The nearest neighbor projection is producing very patchy patterns at low sampling ratios and quickly recovers the ground truth with increased number of samples. In contrast, our proposed method is delivering a good compromise between fine-grained details and coarse-grained interpolation. It is worth noting that when providing very few endocardial samples (0.2 samples/cm^2^) the network is predicting activation times similar to the computational modeling result, suggesting that the proposed method was able to learn a set of modeling priors from the synthetic training set. However, the method is not forced to adhere to the modeling priors of the computational model, thus it is able to deviate from the position of earliest activation. For instance, in Figure 6 one can see that the earliest activation when providing 0.2 samples/cm^2^ is located more anterior compared to the personalized computational model. An additional early activation zone can be recovered in the mid of the anterior wall when providing more samples.

**Figure 7:**
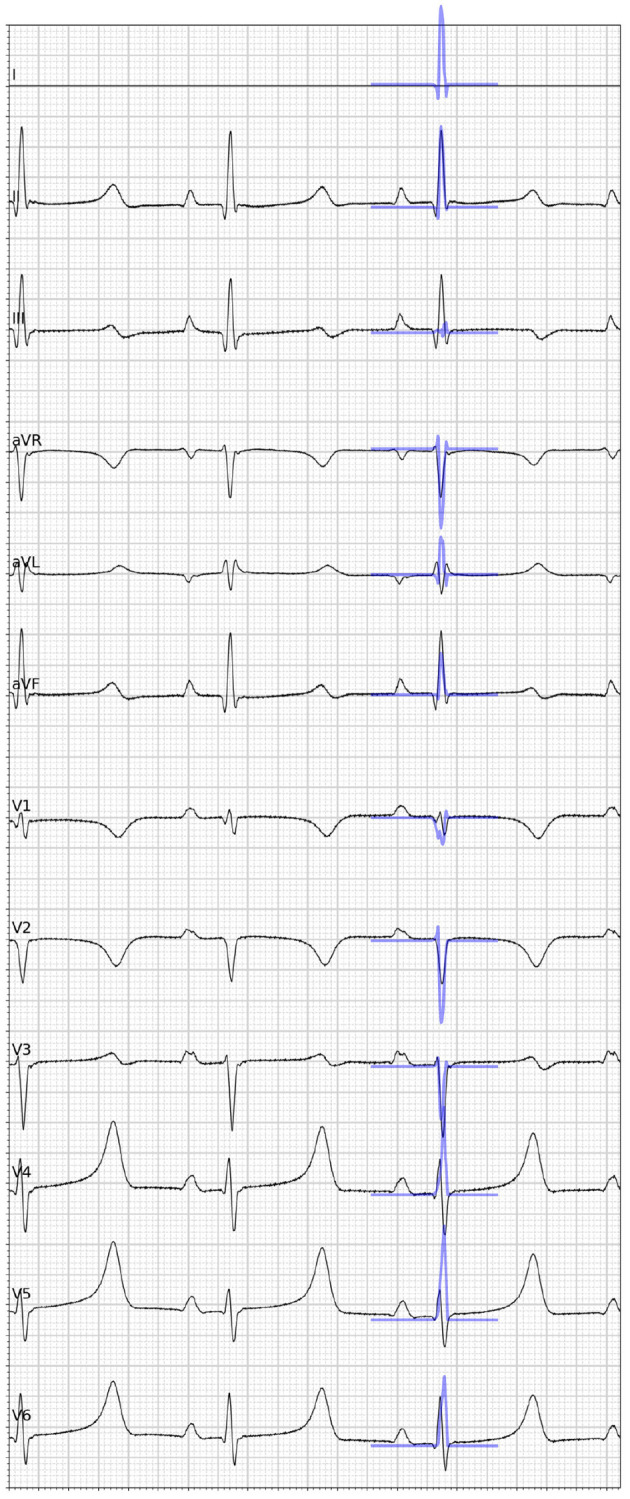
Visual comparison of the ground truth 12-lead ECG (black) taken from one case of the in-house dataset and the synthetic ECG (blue) simulated from the graph convolutional neural network prediction given the full electroanatomical map. Please note that lead I is missing because of a hardware failure.

In addition to the comparison to the measured data, synthetic 12-lead ECGs were computed from the graph convolutional network predictions by calculating intra- and extracellular potentials as described in section 2.2. The resulting traces were compared against the ground truth ECGs. Overall, the signal positivity was matched in the majority of the limb leads. We did, however, observe inconsistencies when comparing relative amplitudes (see Figure 7 for an example). The highest discrepancies were always found in the precordial leads. In particular, inconsistent R/S ratios were observed.

### 3.4. Reconstruction of Epicardial Activation Times From Endocardial Measurements

To evaluate the network performance for predictions of activation times beyond the left endocardium, we applied the network without retraining to the CRT-EPIGGY19 challenge data (Camara, 2019). The dataset comprised eleven swine datasets (four training cases and seven testing cases), for which both endocardial and epicardial electroanatomical mapping has been performed with intrinsic activation after LBBB induction as well as after cardiac resynchronization therapy (CRT). Furthermore, information of the scar extent based on the 17 segment LV model as well as its transmurality was provided. Additional details of the data have been described in Soto Iglesias et al. (2017). For this experiment, we used the endocardial map with intrinsic activation at different subsampling rates. We considered the full endocardial activation map and the epicardial one as the ground truth. To be consistent with the mesh resolution of the training geometries, all tetrahedral meshes provided by the challenge organizers were resampled for an average edge length of 2.5 mm using the CGAL library (The CGAL Project, 2021). Since no ECG traces were provided as part of the challenge, we generated synthetic ECG traces using the following approach. First, the previously used porcine torso (see Figure 4) was rigidly aligned to the tetrahedral meshes by establishing point correspondences. Next, intra- and extracellular potentials were computed from the ground truth activation times as described in section 2.2. This corresponds to the estimated electrical activity during the QRS interval. Synthetic 12-lead ECG traces were then computed from the potentials using the boundary element method as described in Zettinig et al. (2014).

**Figure 8:**
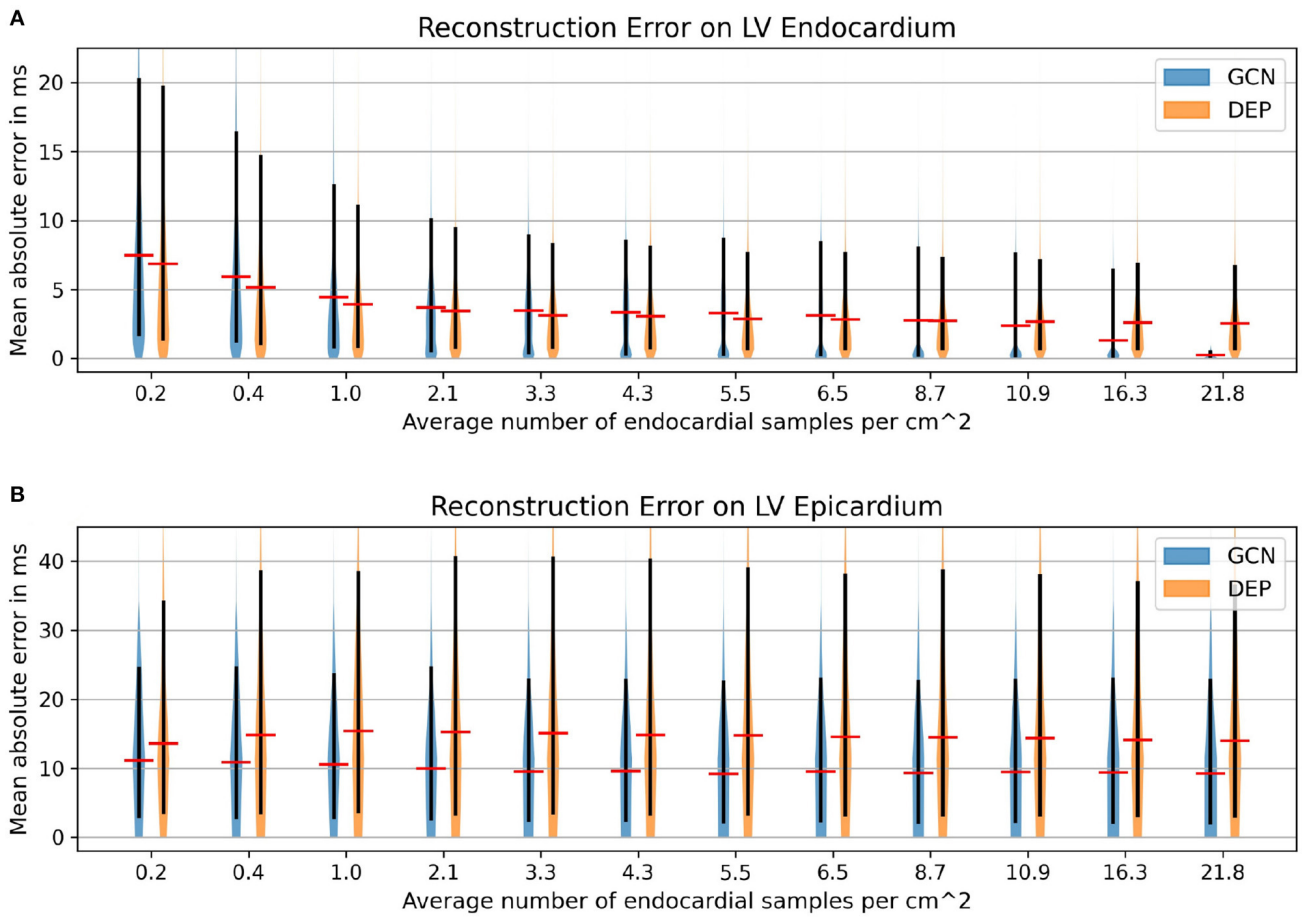
Mean absolute error distributions (mean: red; 15–95 percentile: black) on the left ventricular endocardium **(A)** and the epicardium **(B)** of the graph convolutional predictions on cohort #2 comprising eleven swine datasets with high-resolution endocardial EAMs.

**Figure 9:**
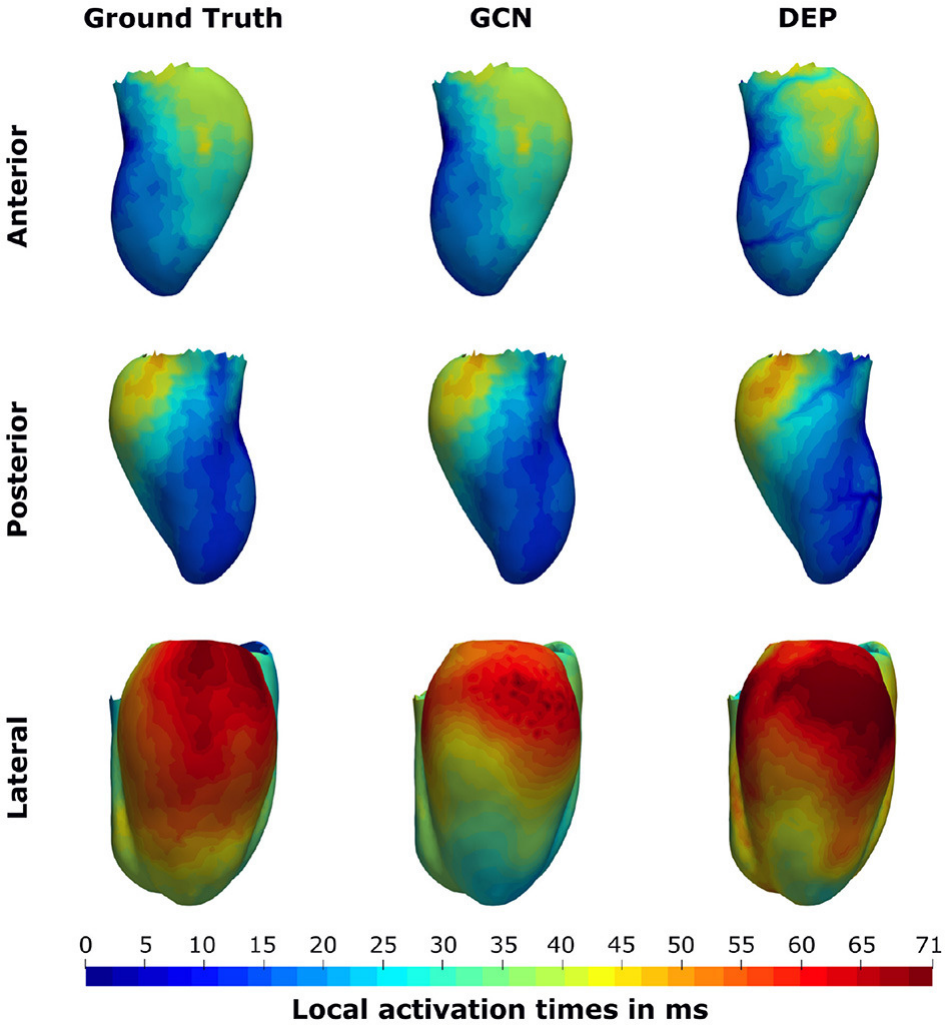
Illustration of the prediction results for the pig “Neus” from the CRT-EPIGGY19 challenge when providing 100% of the endocardial measurements to the graph convolutional neural network (GCN) and the personalized computational model (DEP). The neural network is able to retain the information on the endocardium and provide a coarse approximation of the left epicardial activation time. The computational model fails to accurately match the endocardial information and over-estimates the late activation on the left epicardium.

We compare the network to the electrophysiological model personalized to the same subsampled data as done in the previous section, ranging from 1 to 100% of the endocardial samples (equal to 0.2–21.8 samples/cm^2^). As illustrated in Figure 8A, both the neural network and the personalized computational model are able to reconstruct the entire endocardial map reliably. The neural network is improving more with increased number of endocardial samples and is able to achieve lower errors at sampling ratios of 16.3 samples/cm^2^ and more. When evaluating the performance on the left epicardium (see Figure 8B), both methods produced significantly larger errors with the neural network outperforming the computational model. We observed no significant improvement with increased number of endocardial samples. To measure the overall fit of the prediction to the ground truth measurements (endocardial and epicardial), we follow the approach by Cedilnik and Sermesant (2019) and compute the average over case-wise root median squared errors (RMSE) on the training and test set, respectively. Errors between 5.6 and 7.8 ms were measured for the network for different sampling ratios, with 6.6–9.3 ms for the personalized computational model (see Table 3). Cedilnik et al. reported on the training set a mean RMSE between 9 and 17 ms depending on the personalization scheme of an Eikonal model to both endocardial and epicardial measurements (Cedilnik and Sermesant, 2019). Furthermore, when comparing qualitatively the epicardial ground truth to the solutions provided by the two methods (see Figure 9), the two methods provided very similar wave propagations. They were, however, only coarsely approximating the measured data.

**Table 3:** Comparison of the proposed neural network (GCN) and personalized computational model (DEP) on the training and the test cases of the CRT-EPIGGY19 challenge.

**Data split**	**Training (4 pigs)**			**Testing (7 pigs)**		
**∅ Samples / cm^**2**^**	**6.5**	**16.3**	**21.8**	**6.5**	**16.3**	**21.8**
GCN (Mean RMSE in ms)	6.7	6.2	5.6	7.8	7.3	6.7
DEP (Mean RMSE in ms)	6.8	6.6	6.6	9.3	8.7	8.7

*The mean over per-case root median squared errors (RMSE) is presented for different sampling ratios. RMSE is computed over the vertices with endocardial or epicardial measurements*.

### 3.5. Ablation Study on ECG Features

The preceding experiments leverage ECG information in addition to the measured activation times to estimate biventricular activation maps. In particular, 14 surrogate metrics derived from 12-lead ECG traces (see Table 1) are used as input features to the graph convolutional neural network. This work relies on surrogate metrics since ECG traces may not be present as a digital recording, which would allow automatic feature extraction. Furthermore, ECG traces often exhibit high frequency noise, which could pollute ECG-based features provided as input to the network. The metrics proposed in this work are expected to be less affected by this kind of noise. Nonetheless, it is important that the model learns the relative importance of all provided input features, not relying solely on ECG information. To study the impact of the ECG features on the prediction accuracy, we performed an ablation study. To this end, we trained four additional graph convolutional networks on the same data split as described in section 3.1 and without changes to the hyperparameters (see section 2.1.5). While all networks used the same endocardial measurements and geometry features as input, the ECG information was provided in four different ways: no ECG information, QRS duration only, QRS duration with electrical axis, and QRS duration with vertical positivities.

**Figure 10:**
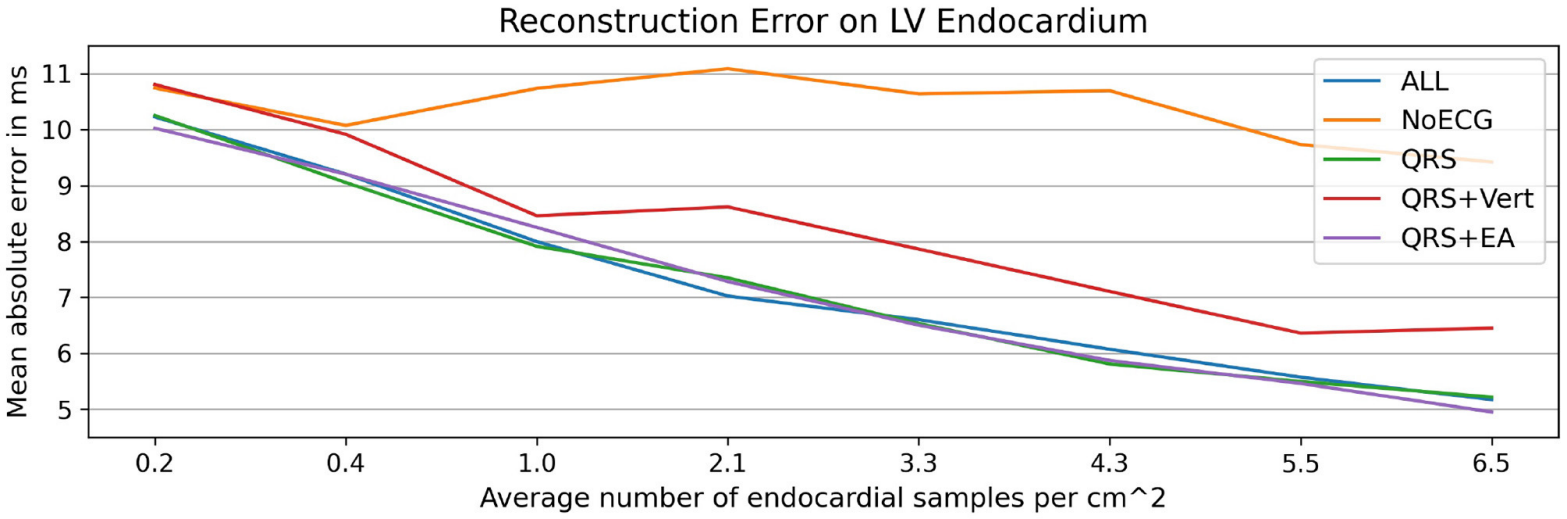
Illustration of the results of the ablation study applied to the in-house dataset. Mean absolute endocardial reconstruction errors are compared for different graph convolutional networks trained on subsets of all features (ALL): no ECG features (NoECG), only QRS duration (QRS), QRS duration with vertical positivities (QRS + Vert), and QRS duration with electrical axis (QRS + EA). The results suggest that ECG information, particularly the QRS duration, is necessary for the accurate estimation of activation maps. The small differences between the networks with ECG features suggest that the networks do not rely solely on ECG features to estimate the endocardial activation maps.

All networks were evaluated on the in-house dataset as described in section 3.3 and compared against the graph network using the original input features. The largest errors were observed when no ECG information was provided, suggesting that ECG information contributes significantly to the reconstruction of the activation map (see Figure 10). The results further show that the QRS duration is the most important ECG feature since its addition leads to comparable performance to the original model. Further addition of electrical axis or vertical positivity leads to improvement in the prediction accuracy only in some of the experiments. This seems to suggest that those features have the potential to contribute to more accurate estimation of the activation times, but their correlation to the ground truth is comparatively less strong than that of other features.

### 3.6. Active Suggestion of New Sampling Locations

The previous experiment demonstrates that ECG information is important for the estimation of activation times. Compared to the steep error reduction with an increase in provided measurements, the small differences between the networks with different ECG features confirm that the method prioritizes information from the measurements. We hypothesize that targeted selection of the measurement locations could boost the performance compared to randomized sampling. To this end, we run an experiment in which new sampling locations are suggested based on the intrinsic uncertainty, measured by the disagreement between the predictions of an ensemble of neural networks, as successfully demonstrated by Sahli Costabal et al. (2020). While Costabal et al. trained multiple networks with different initial conditions and had to repeatedly fine-tune the models with each new sample collection, this work relies on an ensemble of pre-trained networks comprising the four graph convolutional networks from the previous section. Since these models are able to incorporate the given measurements without a re-training step, new samples can be suggested in real-time.

To sequentially propose new sampling locations, the following approach was performed. Starting off with a set of randomly sampled positions, the feature matrices for each network were assembled. Next, each network estimated local activation times. The mean activation map of the four networks as well as vertex-wise standard deviations were computed. Since the standard deviation represents the disagreement between the predictions, the proposal of a new sampling location was chosen to be the vertex location with highest standard deviation. A constraint was added such that each vertex can only be selected once.

To demonstrate that such relatively simple uncertainty estimation is of value, the following scenario was considered. First, the endocardia of the test datasets from the second experiment (see section 3.3) were split into septal regions and free wall regions by applying a vertex-wise threshold of ±90 degree to the angular feature of the local coordinate system. Next, 10% of the free wall vertices were randomly chosen as an initial mapping. The active sampling strategy was then applied only to the septal region. This setup was chosen to study whether new sampling locations will correlate with the presence of scar and border zone, which is located on the septum in all cases of the in-house dataset. For each new sample, the mean activation map was computed from the model ensemble, and the mean absolute error with respect to the ground truth was evaluated. For comparison, a random sampling strategy was applied to three alternative approaches: the graph convolutional network with all features, the personalized computational model, and the nearest neighbor projection.

**Figure 11:**
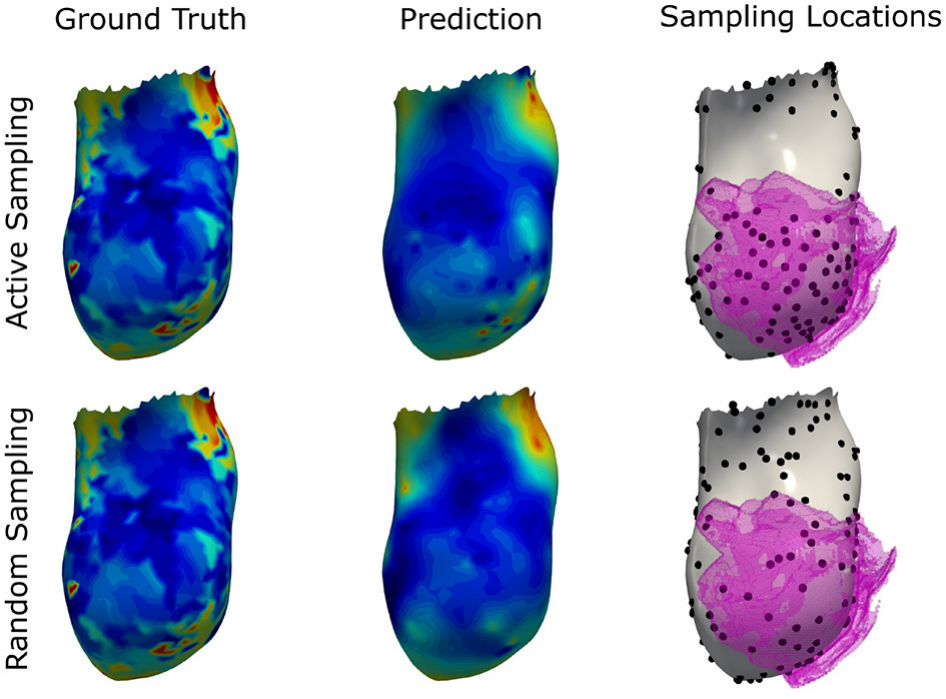
Illustration of the prediction results for the graph convolutional neural network using the proposed active sampling strategy and the random sampling strategy after selecting 10% of septal vertices. Provided samples are highlighted by black dots. The segmented border zone mask is overlayed in pink and located antero-septal in this swine model of myocardial infarction. Compared to the random sampling strategy, the active sampling approach better captures important details of the ground truth, such as the location of earliest activation and deceleration zones associated with the slow conductive border zone.

The results as presented in Table 4 show that the active sampling strategy is producing the lowest error for all subsampling ratios. In particular for very low sampling densities, high differences between the active ensemble and the personalized model as well as the nearest neighbor projection were found. For instance, at 1% the GCN with active sampling agreed with the ground truth with a mean absolute error of 8.5 ms ± 7.9 ms. The personalized computational model and the nearest neighbor projection recovered the ground truth with 9.5 ± 8.9 ms and 14.9 ± 11.4 ms, respectively. Using the random sampling strategy, all methods achieved the lowest errors at a sampling density of 10%. In contrast, the ensemble-based active sampling strategy can achieve the same error with significantly less measurements. For the GCN a reduction by 40% of septal samples was observed, while the reduction for the nearest neighbor method measured 20%. At 10% of septal samples, the estimated activation maps of the active sampling strategy shows a qualitatively better agreement with the ground truth compared to the random sampling result (see Figure 11). For instance, the mid-septal location of earliest activation area found in the ground truth is fully recovered by the active sampling, while the random sampling leads to multiple areas of earliest activation. Moreover, deceleration zones within fast conductive early activated areas, potentially associated with presence of border zone, are fully recovered with the proposed sampling strategy. A visual comparison of the sampling locations and of the segmented border zone indeed shows higher sampling densities in this area. The results are therefore particularly encouraging since the proposed sampling strategy samples in areas that are known for their uncertain tissue characteristics.

**Table 4:** Mean absolute errors (± the standard deviation) in ms between predicted and ground truth activation times at different subsampling ratios of the septal ground truth.

**Subsampling ratio (%)**	**GCN-active**	**GCN-random**	**DEP-random**	**NN-random**
1	8.5 ± 7.9	8.7 ± 8.1	9.5 ± 8.9	14.9 ± 11.4
2	8.3 ± 8.0	8.4 ± 8.2	9.1 ± 8.6	13.7 ± 11.5
3	8.0 ± 7.9	8.3 ± 8.3	9.3 ± 8.4	9.7 ± 10.1
4	7.8 ± 7.9	8.0 ± 8.0	9.0 ± 8.3	9.3 ± 9.5
5	7.5 ± 7.7	7.9 ± 8.0	8.7 ± 8.1	9.3 ± 9.6
6	7.4 ± 7.6	7.7 ± 7.7	8.6 ± 8.1	8.7 ± 9.3
7	7.2 ± 7.5	7.6 ± 7.7	8.5 ± 8.1	8.1 ± 9.4
8	7.1 ± 7.4	7.7 ± 7.7	8.4 ± 8.0	7.4 ± 9.4
9	7.0 ± 7.4	7.5 ± 7.6	8.3 ± 7.9	7.2 ± 9.4
10	7.0 ± 7.4	7.4 ± 7.5	8.2 ± 7.8	7.1 ± 9.4

*Active sampling based on an ensemble of four GCNs (GCN-active) is compared against three methods with random sampling: the graph convolutional network (GCN-random), the personalized computational model (DEP-random), and the nearest neighbor projection (NN-random)*.

## 4. Discussion

This study investigates the data-driven interpolation and extrapolation of sparsely measured left endocardial activation times. A system, capable of estimating the electrical activity from sparse measurements, ECG information, and imaging data, could impact computational cardiology in numerous ways. For instance, the procedural time of the mapping procedure could be significantly reduced since less samples would need to be acquired. Other applications include the suggestion of valuable sampling locations. Paving the way toward this goal, this work relies on a graph convolutional neural network that enables learning to encode topological structure and local features as well as the propagation of information on graph-structured data. Its usage naturally adapts to cardiac electrophysiology, where the electrical wave propagation is directly linked to the structure of the cardiac conduction system, for which graph-based representations are suitable. A synthetic dataset to train the network has been generated by a computational model to overcome the absence of ground truth activation times in the entire biventricular geometry. A statistical shape model as well as randomized scar and border zone distributions, varying conduction velocities, and different initial activation locations have been used to induce variability in the simulations.

Without re-training, the proposed approach was evaluated on unseen synthetic simulations and two real world datasets. In all setups, the graph convolutional neural network was able to accurately reconstruct the endocardial activation maps even when sparse data points were provided. Moreover, decreasing errors were observed with an increase in the number of provided measurement samples, suggesting that the network can incorporate the measured information. Compared to the qualitative results from our previous work (Meister et al., 2021), we observed significantly better agreement with the ground truth activation maps, which we attributed to a better training dataset. In particular, the interpolation of the data appeared to be physically plausible, while the previous work incorporated the measured data as local discontinuities in the predicted activation map. The resulting activation maps were patchy and high accuracy was only achieved in vertices with provided measurements.

Since measuring intramural activation times is commonly infeasible in patients, the method was evaluated only on the left out synthetic data. Here, a good agreement between the ground truth and the prediction was observed. The errors decreased the more endocardial samples were provided to the network. Since the border zone conduction velocity was independently varied and was always connected to the endocardial surface in this cohort, the result suggests that the network was able to account for the presence of intramural border zone based on the provided measured activation times. For the epicardial activation times, the method was evaluated on the CRT-EPIGGY19 challenge. While observed errors were in general significantly higher compared to the other benchmarks, macroscopic features of the epicardial activation map could be reproduced by the network. For instance, the area of latest activation in both measured and estimated activation map was basal with a small shift toward the anterior side (see Figure 9 for comparison). It is important to notice that the measurement of epicardial activation times could be affected by significant sources of noise such as far field effects, motion, and thin epicardial layers of fat, or due to errors in the alignment (van Huls van Taxis et al., 2013; Josephson and Anter, 2015). This could explain at least in part the higher estimation errors on the epicardium. Similar differences were observed by the challenge participants Cedilnik et al., who personalized an Eikonal model to both endocardial and epicardial measurements (Cedilnik and Sermesant, 2019). The authors reported a mean RMSE between 9 and 17 ms depending on the used personalization strategy. In comparison, our graph network achieved a mean RMSE between 5.6 and 7.8 ms depending on the subsampling ratio. The results suggest that the proposed method provides more accurate results compared to Cedilnik et al. even at low subsampling ratios. This holds true despite one intrinsic limitation of our approach, since our model assumed that early activation starts either septal or in the left ventricular endocardium. The challenge data had cases with early activation in the right ventricle. For those cases, the RMSE reported above will be inflated by higher errors in the estimation of the activation times in the right ventricle. We hypothesize that training the network on additional synthetic examples with early right ventricular activation would further improve the results.

We further performed an ablation study to investigate the impact of the ECG features on the prediction accuracy. The results highlight that the inclusion of ECG information, particularly the QRS duration, is important to reconstruct the activation times. Additional features, such as the electrical axis and vertical positivities, only improved the results in some experiments. We hypothesize that these features have the potential to improve predictive accuracy, but the trained model did not rely on them as much as on the other input features. This could be due to the fact that the relationship between these specific metrics (electrical axis and vertical positivity) and the pattern of activation times are intrinsically complex, with potentially different activation maps being associated to ECG signals with analogous surrogate metrics. Usage of more complex features derived from the ECG signals could help the model discriminate better their predictive role. In our animal datasets we observed that the ECG signals were affected by noise in some of the leads, which could make the extraction of the ECG features more challenging. Even though the surrogate metrics are by design less impacted by noise in the measurements, the networks were trained on simulated and thus noise-free data. Investigating data augmentation strategies that add realistic noise to the training data is an interesting area of future research, which may improve the generalization between synthetic and measured data.

In addition, the method was compared against two other methods. When comparing the graph network against a nearest neighbor projection on the task of reconstructing high resolution endocardial activation maps, the data-driven approach outperformed the projection method for low sampling densities. The projection method generated coarse and patchy activation maps, which suggests this method to be sensitive to measurement noise. In contrast, the graph network produced a smooth and physically plausible interpolation of the provided measurements. The qualitative results are comparable to those obtained by the computational model of cardiac electrophysiology, which was personalized to the same provided measurements and which was used as the other comparator. The computational model was, however, not able to capture the intricate details of the high-resolution activation maps due to the strong regularizing effect of the modeling priors. When comparing both methods on the challenge data, the network produced similar or better results than the computational model when attempting to reconstruct the complete endocardial activation map from a sparsefied version. When targeting the estimation of left epicardial activation maps from the endocardial measurements, the network significantly outperformed the computational model. Visually, both methods provided similar epicardial wave front estimations on the challenge data, but the network proved better at capturing the total activation time (see Figure 9).

At last, the feasibility of supporting the mapping procedure by actively suggesting new sampling locations was investigated. Using an ensemble of graph neural networks, the disagreement between predictions was used to determine locations of high uncertainty, which were then used as new sampling locations. The *in-silico* study on the in-house dataset suggests that this rather simple approach was able to suggest meaningful new sampling locations that lead to lower errors compared to random sampling of the same number of points. To match the same accuracy as when providing 10% randomly selected septal measurements as input to the graph network, a reduction of up to 40% sampling points was achieved with the active sampling strategy, which could result in a significant reduction of the mapping procedure duration. Compared to a similar synthetic experiment, which used physics-informed neural networks and relied on fine-tuning of a network ensemble for each new sample (Sahli Costabal et al., 2020), our method showed a significant runtime advantage since it was able to directly integrate newly measured data points. Up to 2 samples per second could be processed with this approach. In contrast, Costabal et al. reported that fine-tuning alone took approximately 1 min per sample (Sahli Costabal et al., 2020). Furthermore, not only the endocardial activation times were estimated, but local activation times in the entire biventricular domain. Additional speed gains are expected with further algorithmic and architectural optimizations.

While the present study demonstrated a new way for joint interpolation and extrapolation of measured activation times, the proposed method presents several limitations. First, a noticeable difference between the observed errors on the synthetic and measured data was found. The higher errors may be explained by the high amplitude noise in the measured data, which is particularly present in the in-house dataset (see Figure 6 for an example). Since the noise manifests as seemingly unphysical local discontinuities, the proposed approach could be regarded as a physics-inspired denoising procedure. This however would have to be demonstrated by comparing the model results against a larger set of measured maps with varying signal-to-noise ratio. Furthermore, we hypothesize that the proposed method is highly dependent on the underlying computational model used to generate the training dataset. This is supported by the qualitatively similar activation maps that were produced by the graph network and the computational model, particularly for very low sampling densities. The training data used in this work comprised simulations with limited variability in modeling assumptions. For instance, the use of 180 geometries sampled from a statistical shape model computed from only 11 swine datasets could be too limiting to cover the wide range of anatomical variability. In the experiments conducted in this study we did not observe a significant variation of the error metrics across different anatomical models in the testing set. Nonetheless, the relatively small sample size in our experiments does not allow to generalize this finding and a richer statistical shape model could make the trained network more robust to geometry variations. Furthermore, scar and border zone were modeled by a relatively simple heuristic that results in primarily transmural distributions. This modeling assumption is particularly suitable for severely scarred cases, such as the swine hearts considered in this work. Applying our approach to more general cases, such as intricate intramural channels within core scar, may require further enrichment of the training dataset.

The graph representations adopted in this work were based on tetrahedral approximations of the biventricular heart geometry, with a relatively coarse mesh resolution (2.5mm). This implies that the spatial distribution of activation times in the tissue (both in the training and in the testing sets) is approximated and does not include fine spatial details at lower scales. This could potentially affect the capability of the model to accurately represent transmural gradients in activation times. This, however, is expected to be acceptable in the modeling of swine hearts, in which transmural gradients of activation time are relatively small due to the transmural Purkinje tree (Garcia-Bustos et al., 2017). Considering graphs with relatively large edge length has the advantage that the graph convolutional network architecture can be more compact, requiring less convolutional layers to represent the same receptive field. The increased number of convolutional layers required by graphs with shorter edge lengths was reported to potentially degenerate the network performance due to over-squashing and over-smoothing (Alon and Yahav, 2020). Making graph convolutional neural network invariant to the underlying mesh resolution is still an understudied problem and an interesting direction for future research.

Our model was based on the assumption that the heart tissue can be represented as the union of five regions, each characterized by homogeneous and constant conduction velocity. In particular, the transmural Purkinje network found in swines was simply approximated by a 3 mm layer for the left and right endocardium, respectively. In addition, we also assumed that sites of initiation of the ventricular depolarization are known and the same for all hearts. The results under these modeling assumptions showed good agreement between estimated and ground truth activation maps. It is, however, possible that less restrictive or more complex modeling assumptions would increase the fidelity of the underlying electrophysiology model. Similarly, this work employed an Eikonal solver of cardiac electrophysiology. While the solver proved suitable to represent non-arrhythmic cardiac activation, it may fail to capture wave re-entrants. Further, our graph convolutional approach appeared to be influenced by the fidelity of the underlying training data, which is limited by the previously mentioned modeling assumptions. An interesting future research direction hence comprises the application of more complex cardiac electrophysiology solvers together with higher fidelity modeling assumptions for the training data generation, which may in turn improve the accuracy of the network estimations even in non-sinus rhythm.

The proposed method further integrated information from 14 ECG features comprising the QRS duration, electrical axis, and the relative amplitude of each lead. The results of our ablation study suggest that the ECG features, particularly the QRS duration, are important to estimate activation maps. It is possible that such surrogate metrics hide details of the ECG signals that could improve the predictive performance. Also in this case, access to large quantities of preferably noise-free datasets with digitalized ECG recordings is required to investigate the role of full trace information. Similarly, it would be necessary to acquire precise information about the lead placement and the exact heart-torso orientation to investigate the observed discrepancies between the ground truth and synthetically generated ECG traces as seen in Figure 7.

Another interesting direction to potentially further improve the proposed method is the investigation of more elaborate network architectures. For instance, alternative graph convolutional filter were explored in the literature, which have the advantage of learning more expressive feature representations and may allow the incorporation of edge information (Wu et al., 2020). In the context of the proposed method, they could be easily integrated as a replacement for the SAGE layers of the local feature extractor and may help improve the agreement between model predictions and ground truth activation time, particularly in graph nodes in which the measurement is provided as input feature.

In addition, we strongly believe that acquiring large amounts of high quality datasets will be critical to further improve the proposed approach. It would help clarifying what is the role of each source of noise or uncertainty, both from the data and the modeling assumptions. For instance, we hypothesize that more elaborate scar and border zone models, potentially built from a large set of image data, as well as inhomogeneous tissue conduction velocities could lead to higher fidelity in the training dataset. To this end, measurements with high signal to noise ratio would help elucidate which modeling assumptions are of particular relevance. Furthermore, we hypothesize that such information could strengthen the physics-inspired denoising capabilities of the proposed method.

The same improvements of the modeling assumptions and ECG features as described above may also increase the performance of the personalized computational model. While the computational model was able to estimate the main mode of electrical wave propagation, it did exhibit a stronger regularizing effect compared to the other two methods. Possible areas of improvement may comprise the personalization of initiation sites and constraining the fine-tuning process with ECG information. Alternatively, personalization strategies that include full ECG traces instead of surrogate metrics, such as presented in Gillette et al. (2021) and Pezzuto et al. (2020), could be employed. Another interesting area of future research may include the incorporation of uncertainties related to the ECG generation such as the uncertainty in lead placement.

## 5. Conclusion

This work proposes a deep learning based approach to estimate biventricular local activation times given a spatial discretization of cardiac anatomy, i.e., a tetrahedral mesh, a routinely acquired 12-lead ECG, and sparse endocardial activation time measurements. In particular, the use of graph convolutional layers is explored, which allows the propagation of information through the mesh structure. In total, 24 geometric and electrophysiological features are used for the prediction task. Due to data scarcity and no ground truth information for the intramural activation times, a synthetic training dataset is generated using a fast graph-based computational model of cardiac electrophysiology with depolarization variations induced by randomized conductivities, randomized scar and border zone distributions, variable initial activations and variable geometries from a statistical shape model. The proposed method has been validated using 870 left out simulations and two clinical cohorts with a total of 15 swine datasets. Good agreement between the endocardial ground truth activation times and the predictions have been observed in all setups. Compared to a personalized computational model the proposed approach is producing similar or better results, while not requiring a time-consuming iterative personalization process. Further research is required to assess and improve the moderate agreement between epicardial prediction and the ground truth of the clinical cohort.

## Data Availability Statement

The data analyzed in this study is subject to the following licenses/restrictions: The in-house dataset cannot be shared due to contractual obligations - contact: Henry Halperin, hhalper@jhmi.edu. The CRT-EPIGGY19 challenge dataset is publicly available. Requests to access the data should be directed to the CRT-EPIGGY19 challenge (homepage: https://crt-epiggy19.surge.sh; data: https://zenodo.org/record/3249511; contact: Oscar Camara, oscar.camara@upf.edu).

## Ethics Statement

The animal study was reviewed and approved by the Institutional Review Board and Ethics Committee at Hospital Clínic de Barcelona, Spain (approval reference number: DMAH 5648) for the CRT-EPIGGY19 challenge; and the Johns Hopkins Animal Care and Use Program (ACUC), 1620 McElderry Street; Reed Hall, Room B122; Baltimore, MD 21205-1911 for the in-house dataset.

## Author Contributions

FM gave impulse to the idea, conducted all experiments and evaluations, and wrote the majority of the manuscript. TM, TP, and FM refined the core idea. TM, TP, AM, CA, ÈL, and VM provided continuous feedback and ideas during the experimentation phase. HH and HA provided clinical data and expertise. TM, HH, TP, AM, CA, ÈL, VM, and HA supported the writing process with feedback and local refinements. The manuscript was reviewed by all authors.

## Funding

HA was supported by a grant from Fondation Leducq (Grant Number 16CVD02).

## Author Disclaimer

This feature is based on research, and is not commercially available. Due to regulatory reasons its future availability cannot be guaranteed.

## Conflict of Interest

TP, CA, ÈL, VM, and TM are employees of Siemens Healthineers. FM's research is funded by Siemens Healthineers. The remaining authors declare that the research was conducted in the absence of any commercial or financial relationships that could be construed as a potential conflict of interest.

## Publisher's Note

All claims expressed in this article are solely those of the authors and do not necessarily represent those of their affiliated organizations, or those of the publisher, the editors and the reviewers. Any product that may be evaluated in this article, or claim that may be made by its manufacturer, is not guaranteed or endorsed by the publisher.
